# Alterations of Innate Immunity Reactants in Transition Dairy Cows before Clinical Signs of Lameness

**DOI:** 10.3390/ani5030381

**Published:** 2015-08-14

**Authors:** Guanshi Zhang, Dagnachew Hailemariam, Elda Dervishi, Qilan Deng, Seyed A. Goldansaz, Suzanna M. Dunn, Burim N. Ametaj

**Affiliations:** Department of Agricultural, Food and Nutritional Science, University of Alberta, Edmonton, AB T6G 2P5, Canada; E-Mails: guanshi@ualberta.ca (G.Z.); hailemar@ualberta.ca (D.H.); eda_d2001@hotmail.com (E.D.); qilan@ualberta.ca (Q.D.); goldansaz@ualberta.ca (S.A.G.); suzanna1@ualberta.ca (S.M.D.)

**Keywords:** dairy cow, blood, cytokines, acute phase proteins, metabolites, lameness

## Abstract

**Simple Summary:**

Lameness is prevalent in dairy cows and early diagnosis and timely treatment of the disease can lower animal suffering, improve recovery rate, increase longevity, and minimize cow loss. However, there are no indications of disease until it appears clinically, and presently the only approach to deal with the sick cow is intensive treatment or culling. The results suggest that lameness affected serum concentrations of the several parameters related to innate immunity and carbohydrate metabolism that might be used to monitor health status of transition dairy cows in the near future.

**Abstract:**

The objectives of this study were to evaluate metabolic and innate immunity alterations in the blood of transition dairy cows before, during, and after diagnosis of lameness during periparturient period. Blood samples were collected from the coccygeal vain once per week before morning feeding from 100 multiparous Holstein dairy cows during −8, −4, disease diagnosis, and +4 weeks (wks) relative to parturition. Six healthy cows (CON) and six cows that showed clinical signs of lameness were selected for intensive serum analyses. Concentrations of interleukin-1 (IL-1), interleukin-6 (IL-6), tumor necrosis factor (TNF), haptoglobin (Hp), serum amyloid A (SAA), lipopolysaccharide binding protein (LBP), lactate, non-esterified fatty acids (NEFA), and β-hydroxybutyrate (BHBA) were measured in serum by ELISA or colorimetric methods. Health status, DMI, rectal temperature, milk yield, and milk composition also were monitored for each cow during the whole experimental period. Results showed that cows affected by lameness had greater concentrations of lactate, IL-6, and SAA in the serum *vs.* CON cows. Concentrations of TNF tended to be greater in cows with lameness compared with CON. In addition, there was a health status (Hs) by time (week) interaction for IL-1, TNF, and Hp in lameness cows *vs.* CON ones. Enhanced serum concentrations of lactate, IL-6, and SAA at −8 and −4 wks before parturition were different in cows with lameness as compared with those of the CON group. The disease was also associated with lowered overall milk production and DMI as well as milk fat and fat-to-protein ratio. In conclusion, cows affected postpartum by lameness had alterations in several serum variables related to innate immunity and carbohydrate metabolism that give insights into the etiopathogenesis of the disease and might serve to monitor health status of transition dairy cows in the near future.

## 1. Introduction

Lameness is the third most prevalent disease in dairy herds after infertility and mastitis that contributes to economic loss to dairy farmers [1]. Harmful effects of lameness include lower milk yield [2] and reproductive performance [3], and increased involuntary culling rates [4]. Lameness is a very costly disease that has been estimated to cost producers between $121 and $216 per case [5]. Lameness is often difficult to detect before appearance of clinical signs of disease and by the time it is detected it is often very difficult or too late to treat or even save the cow. Early detection and treatment can improve the animal well-being, improve recovery rate, increase longevity, and minimize financial losses.

Lameness can be broadly categorized into two types: non-infectious lameness and infectious lameness [5]. The etiopathology of different types of lameness is not fully understood and various hypotheses have been suggested in the past. For example Bergsten *et al.* [6] indicates that feeding large amounts of grains is highly associated with laminitis-related lameness. Elevated concentrations of starch in the rumen initiate a state of ruminal acidosis. The latter is associated with death of both Gram-negative and Gram-positive bacteria and the release of large amounts of lipopolysaccharide (LPS) and potentially lipoteichoic acid (LTA), which might be involved in the etiology and pathogenesis of laminitis [7].

Various investigators have used pro-inflammatory cytokines and acute-phase proteins (APPs) as biomarkers of lameness [8,9]. Cytokines are proteins produced mainly by macrophages, T-cells, Kupffer cells, and natural killer cells [10,11,12]. One of the major functions of pro-inflammatory cytokines is to stimulate production of acute-phase proteins like haptoglobin (Hp), serum amyloid A (SAA), and lipopolysaccharide binding protein (LBP) [13,14]. Three cytokines including interleukin-1 (IL-1), interleukin-6 (IL-6), and tumor necrosis factor (TNF) have been reported to be the main stimuli for production of APP [12,15,16]. In addition, Hp, SAA, and LBP have been reported as useful variables for assessing the overall health status of domestic animals [17,18].

Presently, there is little information about alterations related to innate immunity reactants or carbohydrate and lipid metabolic profiles in the blood of transition dairy cows before appearance of clinical signs of lameness. In this study, we hypothesized that by measuring selected blood metabolites, pro-inflammatory cytokines, and acute phase proteins (APPs) we would be able to identify blood alterations that could be used to explain the etiopathology of lameness or as early biomarkers of disease in transition dairy cows in the near future. Therefore, the objectives of this investigation were to screen for changes in blood metabolites related to carbohydrate and lipid metabolism and innate immunity starting at −8 or −4 weeks (wks) before the expected day of parturition by measuring the concentrations of serum metabolites like lactate, non-esterified fatty acids (NEFA), β-hydroxybutyrate (BHBA) as well as pro-inflammatory cytokines including IL-1, IL-6, and TNF and APPs like Hp, SAA, and LBP. The same blood variables will be evaluated also during disease diagnosis and after diagnosis of lameness to be used for differential diagnosis and recovery rates.

## 2. Materials and Methods

### 2.1. Animals and Diets

One hundred pregnant Holstein dairy cows at the Dairy Research and Technology Centre, University of Alberta (Edmonton, AB, Canada), were used in a longitudinal study. Six pregnant multiparous (parity: 3.0 ± 0.6, mean ± SEM) Holstein dairy cows were diagnosed with lameness (diagnosed at wk +1, +2, +2, +3, +3, and +3, respectively) and six healthy control cows (CON) that were similar in parity (3.3 ± 0.6), age, and body condition score (BCS), were selected for this nested case-control study. All experimental procedures were approved by the University of Alberta Animal Policy and Welfare Committee for Livestock, and animals were cared for in accordance with the guidelines of the Canadian Council on Animal Care [19].

The experimental period lasted for 17 wks from −8 wks before parturition to +8 wks postpartum (*i.e.*, −8 wks to +8 wks, 0 wk means the week of calving) for each cow. Cows were housed in individual tie stalls bedded with sawdust and with free access to water throughout the experiment. Shortly before calving cows were transferred to the maternity barn and returned to their stalls on the following day of parturition. Diets were offered as TMR for ad libitum intake once daily at 0800 h to allow approximately 5% orts (Table 1 and Table 2). All TMR were formulated to meet or exceed the nutrient requirements of dry and early 680 kg lactating cows as per National Research Council guidelines (2001) [20]. Individual dry matter intake (DMI) was recorded daily throughout the 17 wks period by calculating the difference between the total daily diets given to each cow with the orts on the next morning. Since the onset day of lactation, cows were milked in their stalls twice per day at 0500 and 1600 h, and individual milk yield (MY) was recorded electronically. Milk compositions like crude protein (CP), milk fat, lactose, somatic cell count (SCC), milk urea nitrogen (MUN), and total solids (TS) were analyzed by mid-infrared spectroscopy (MilkoScan 605; A/S Foss Electric, Hillerød, Denmark) at the DHI Central Milk Testing Laboratory in Edmonton, Alberta.

**Table 1:** Prepartum diet for dry cows.

	Close-Up
Item	diet (CUD)
Ingredient	%, DM
Alfalfa hay	10.0
Barley silage	60.0
CUD grain	30.0
Nutrient composition of CUD grain mix	% amount per kg
Ruminant TM Pak ^1^	0.2775
Selenium 1000 mg/kg (UNscr FineCr)	0.2
Custom TM Complex Premix ^2^	0.33
Vitamin A/D_3_-1000-200 ^3^	0.006
Barley grain, rolled	39.5815
Flo-bond mycotoxin binder	0.5
Limestone	3.7
Magnesium chloride	1.645
Mag Ox-56% ^4^	0.54
Scale Molasses (60:40)	2.5
Canola meal	17.0
Vitamin E 50% Ads ^5^	0.18
Soybean hulls, ground	33.0
Salt	0.54

^1^ Ruminant TM Pak: a premix containing cobalt, copper, iodine, manganese, and zinc. ^2^ Custom TM complex premix: a custom product supplying organic sources of cobalt, copper, manganese, and zinc. ^3^ Vitamin A/D_3_-1000-2003: Vitamin A acetate (retinyl acetate) and Vitamin D_3_ (cholecalciferol). ^4^ Mag Ox 56%: 56% magnesium guarantee. ^5^ Vitamin E 50% Ads contains 226,800 IU of Vitamin E per pound.

**Table 2:** Ingredients of high grain ration fed to cows during early lactation.

	Early
Item	Lactation Diet
Ingredient % of DM	%, DM
Alfalfa Hay	9.59
Barley Silage	30.24
Alfalfa Silage	9.64
High 16% dairy ration	50.5
Nutrient composition of dairy ration	% amount per kg
ADE Vit Pak-30 Natural E ^1^	0.05
Ruminant TM Pak ^2^	0.11
Selenium, 1,000 mg/kg (UNscr FineCr)	0.07
Custom TM Complex premix ^3^	0.07
AminoShure-L ^4^	0.33
Blood meal	3.50
Barley grain, rolled	39.91
Barley grain, ground	27.50
Di-calcium phosphate 21%	1.00
Vit D-10,000 KIU/kg	0.02
Diamond V XPC ^5^	0.13
Dairy Xtract	0.02
Energizer RP10	2.75
Limestone	1.70
Mag Ox-56% ^6^	0.43
Scale Molasses (60:40)	1.25
Nutri A-Z C Dry	0.10
Amino Plus (High bypass soy) ^7^	8.00
Vitamin E 50% Ads ^8^	0.01
Soy bean meal-47.5%	1.25
Sodium bicarbonate	0.80
Salt	0.51
Poultry-Tallow	0.50
Biotin 2%-Rovimix H-2 ^9^	0.01
Wheat distillers grain (50:50)	10.00

^1^ ADE Vit Pak-30 Natural E: a premix containing vitamins A, D3, and E. ^2^ Ruminant TM Pak: a premix containing cobalt, copper, iodine, manganese, and zinc. ^3^ Custom TM complex premix: a custom product supplying organic sources of cobalt, copper, manganese, and zinc. ^4^ AminoShure-L: hydrogenated vegetable oil, and L-lysine monohydrochloride (Halchemix, Port Perry, ON, Canada). ^5^ Diamond V XPC: concentrated yeast (Diamond V Mills, Cedar Rapids, IA, USA). ^6^ Mag Ox 56%: 56% magnesium guarantee. ^7^ Amino Plus: a high by-pass soy meal. ^8^ Vitamin E 50% Ads contains 226,800 IU of Vitamin E per pound. ^9^ DSM Nutritional Products (Parsippany, NJ, USA).

### 2.2. Monitoring the Clinical Health Status of the Cows

Health status (HS) of cows was monitored daily based on clinical signs of disease by trained individuals and on a weekly basis by a veterinary practitioner. All periparturient diseases and veterinary treatments were recorded for each cow throughout the entire experimental period. Diagnosis of pregnancy was performed routinely by a veterinary practitioner at 60–70 days post-insemination. Based on the artificial insemination (AI) data supported with the information of pregnancy diagnosis, the expected date of parturition was fixed by adding 280 days from the day of AI. All cows were monitored daily starting at −8 wks prior to the expected date of calving and continuing up to +8 wks postpartum. The various external symptoms observed were gait, general appearance, appetite, alertness, rectal temperature, ease of calving, body condition score (BCS), vaginal discharges (color and consistency), udder edema, and pain in the legs.

In this study, lameness was diagnosed by trained staff based on a locomotion score system according to the farm standard operating procedure [21]. All six lameness cows used in this experiment had a score of 5, which showed severe lameness with pronounced arching of the back, reluctant to move, and complete weight transfer off the affected limb. The 6 healthy cows had a lameness score of 1. Cows with lameness were treated by trimming and medication [22]. Lame cows were administered either Excenel^®^ RTU (Zoetis Canada, Kirkland, QC, Canada) at 1 mL per 50 kg IM, once a day for 3 days, or Procaine Penicillin G^®^ (Dominion Veterinary Laboratories Ltd., Winnipeg, MB, Canada) at 2 mL per 45 kg IM twice a day for 3 days.

### 2.3. Sample Collection

Blood samples were obtained from the coccygeal vein once per week at 0700 before feeding from −8 wks before parturition to +8 wks postpartum. All blood samples were collected into 10 mL vacutainer tubes (Becton Dickinson, Franklin Lakes, NJ, USA) and allowed to clot and kept at 4 °C until separation of serum. Clotted blood was centrifuged at 2090× *g* at 4 °C for 20 min (Rotanta 460 R centrifuge, Hettich Zentrifugan, Tuttlingen, Germany). The separated serum was aspirated from the supernatant gradually by transfer pipets (Fisher Scientific, Toronto, ON, Canada) without disturbing the sediment. The separated serum was transferred to a sterile 10 mL plastic test tube (Fisher Scientific, Toronto, ON, Canada). All serum samples were stored at −80 °C until analysis to avoid loss of bioactivity and contamination and were thawed on ice for approximately 2 h before use.

Cows were milked twice per day at 0500 and 1600 h, and milk samples collected on day 0, 14, 21, 35, and 49 relative to parturition (day 0 means the day of calving), were used for the analysis of milk composition including crude protein (CP), milk fat, lactose, somatic cell count (SCC), milk urea nitrogen (MUN), and total solids (TS).

### 2.4. Sample Analyses

#### 2.4.1. Serum Metabolites

Quantitative determination of serum lactate, beta-hydroxy butyric acid (BHBA), and non-esterified fatty acids (NEFA) were measured by an enzymatic colorimetric method using commercially available kits provided by Stanbio Laboratory (Boerne, TX, USA) and Wako Chemicals (Richmond, VA, USA), respectively. The detailed methods have been described previously by Ametaj *et al.* [23]. Briefly, according to the manufacturers’ instructions, the lower detection limits of the kits were 0.06 mg/dL, 0.125 μmol/L, and 0.50 μEq/L, respectively. The principle of the lactate assay involves reduction in the colorless tetrazolium salt by an NADH-coupled enzymatic reaction to formazan, which develops a red color change proportional to the lactate concentration. BHBA test involves the basic principle of conversion of serum BHBA to acetoacetate and NADH by BHBA dehydrogenase in presence of NAD. Then, the NADH reacts with 2-p-iodophenyl-3-p-nitrophenyl-5-phenyltetrazolium chloride (INT) in the presence of diaphorase to form a pink colored adduct proportional to the concentration of BHBA in the serum. The principle of NEFA kit involves acylation of coenzyme A (CoA) by fatty acids in the serum in the presence of acyl-CoA synthetase and production of hydrogen peroxide in the presence of acyl-CoA oxidase. Hydrogen peroxide, together with peroxidase, permits the oxidative condensation of 3-methyl-N-ethyl-N-β-hydroxy ethyl-O-aniline with 4-aminoantipyrine to produce a purple color change, which is proportional to the serum NEFA concentration. All samples were tested in duplicate and absorbance of standards and samples *vs.* a blank for lactate, BHBA, and NEFA were read at 492, 505 and 550 nm, respectively, in a microplate reader (Spectramax 190, Molecular Devices Corporation, Sunnyvale, CA, USA). The intra-assay variation of all the three assays was controlled by CV limits <10%.

#### 2.4.2. Serum Cytokines

Concentration of IL-1 in the serum was assayed by a commercially available bovine ELISA kit (Cusabio Biotech Co. Ltd., Wuhan, China) with mAB specific for IL-1 coated on the walls of the microplate strips provided. The procedure involves the basic principle of a competitive inhibition enzyme immunoassay between biotin-conjugated IL-1 and IL-1 with the pre-coated antibody. All samples (50 μL) were tested in duplicate in microtitration wells with biotin-conjugated IL-1 according to the manufacturer’s instructions. The plates were washed with wash buffer after the incubation for 60 min at 37 °C, followed by addition of 50 μL of horseradish peroxidase (HRP)-avidin. Samples were incubated for 30 min at 37 °C. Then, they were washed three times with buffer, and 50 μL substrate A and 50 μL of substrate B reagent were added to each well. After incubation at 37 °C for 15 min, the resulting color reaction was read at 450 nm by a microplate reader (Spectramax 190, Molecular Devices Corporation, Sunnyvale, CA, USA) within 10 min, and the final IL-1 concentration was calculated using a 4-parameter logistic curve fit. The sensitivity of this assay was 250 pg/mL, and the intra-assay coefficient of variation (CV) was <10%.

Concentration of IL-6 in the serum was measured with a bovine ELISA kit provided by Uscnk Life Science Inc. (Wuhan, China) as described by the manufacturer. The detection limit of the assay was 7.8 pg/mL and the intra-assay variation of all IL-6 assays was controlled by CV limits <10%. The principle of the IL-6 test involves a sandwich enzyme immunoassay, which exhibits a yellow color change proportional to IL-6 concentration. Samples or standards were added to the microtiter plate wells with a biotin-conjugated antibody specific for IL-6 with all samples in duplicate. Then, HRP-avidin were added and incubated. After 3, 3′, 5, 5′-tetramethylbenzidine (TMB) substrate and sulphuric acid solution were added, the color change was measured spectrophotometrically at a wave length of 450 nm (Spectramax 190, Molecular Devices Corporation, Sunnyvale, CA, USA).

Concentration of TNF in the serum was determined by a commercially available bovine ELISA kit (Bethyl Laboratories, Inc., Montgomery, TX, USA) using the method described previously [24]. Briefly, all samples were tested in duplicate and the optical density values were read at 450 nm on a microplate spectrophotometer (Spectramax 190, Molecular Devices Corporation, Sunnyvale, CA, USA). The detection range of TNF assay was between 0.078 and 5 ng/mL, and the intra-assay CV was controlled <10%.

#### 2.4.3. Serum Acute Phase Proteins (APPs)

Methods used for the measurement of concentrations of Hp (Tridelta Development Ltd., Co., Kildare, Ireland), SAA (Tridelta Development Ltd.), and LBP (Hycult Biotech, Uden, the Netherlands) in the serum were described previously in detail [25]. In brief, serum samples for LBP and SAA analyses were initially diluted 1:100 and 1:500, respectively. Samples for Hp were not diluted. The minimum detection limits for Hp, SAA, and LBP assays were 2.5 mg/mL, 18.8 ng/mL, and 1.6 ng/mL, respectively. All samples were tested in duplicate and the optical densities were measured at 600 nm for Hp and 450 nm for both SAA and LBP. The intra-assay variations of all three APP assays was controlled by CV limits no more than 10% and for those greater than 10% samples were reanalyzed.

### 2.5. Statistical Analyses

Multivariate analysis was performed using MetaboAnalyst [26]. Recommended statistical procedures for principle component analysis (PCA) and partial least squares discriminant analysis (PLS-DA) were followed according to previously published protocols [26]. To perform a standard cross-sectional two-group study, we compared healthy cows’ group and lameness cows’ group at each time point (−8, −4, disease diagnosis, and +4 wks).

For parametric analysis of the data ANOVA was used by MIXED procedure of SAS (SAS Institute Inc., Cary, NC, USA, Version 9.2) according to the following model:

Y_ijk_ = μ+ S_i_ + W_j_ + (SW)_ij_ + e_ijk_

where Y_ijk_ is the observations for dependent variables, μ represents the population mean, S_i_ is the fixed effect of health status i (i = 1–2, sick cows compared with healthy control separately), W_j_ is the fixed effect of measurement week j (j = 1–4 or 1–17), SW_ij_ is the fixed effect of health status by week interaction, and e_ijk_ is the residual error.

Measurements taken at different weeks on the same cow were considered as repeated measures in the ANOVA. The variance–covariance structure of the repeated measures was modeled separately for each response variable according to the lowest values of the fit statistics based on the Akaike Information Criteria (AIC), AIC corrected (AICC), and Bayesian information criteria (BIC), and an appropriate structure was fitted. Degrees of freedom were approximated by the method of Kenward-Roger (ddfm = kr).

In order to identify early indicators of lameness, average serum concentrations in the week of diagnosis, −8 and −4 wks before the expected day of parturition were compared using t-test of SAS 9.2 between health controls and cows with lameness. Data are exhibited as least-squares means (LSM) and the respective standard error of the mean (SEM). All statistical tests were two-sided. Significance was declared at *P* < 0.05, and tendency was defined at 0.05 < *P* < 0.10.

To analyze correlations between milk SCC at diseased week and serum parameters in specimens collected at the same time and −8, −4, and +4 wks around calving Pearson correlation coefficient and corresponding *P*-values were calculated using the CORR procedure of SAS 9.2 based on a two-tailed test.

## 3. Results

### 3.1. Serum Metabolites

**Figure 1:**
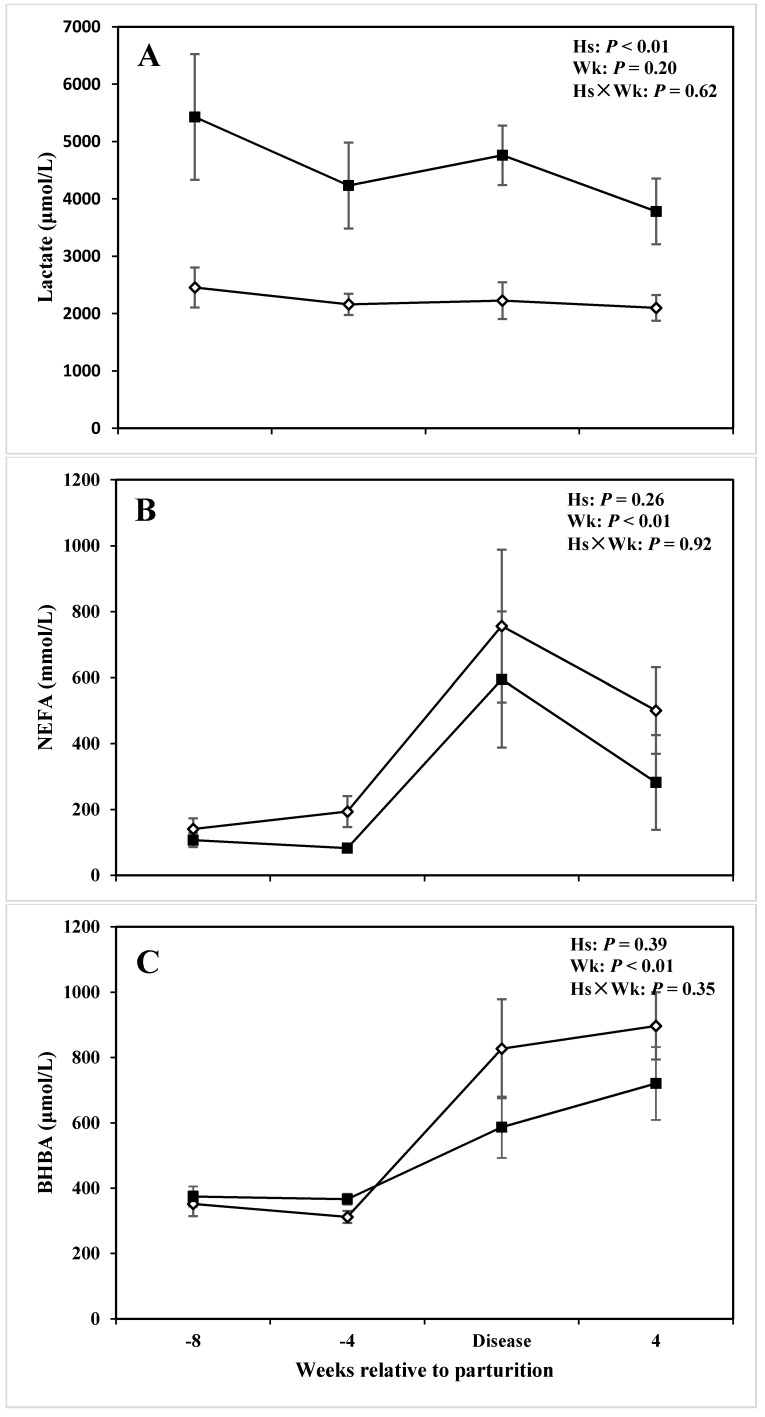
Concentration of (**A**) lactate, (**B**) non-esterified fatty acids (NEFA), (**C**) β-hydroxybutyrate (BHBA) in the serum of periparturient dairy cows with (■, n = 6) or without (◇; n = 6) lameness (LSM ± SEM; Hs = effect of health status; Wk = effect of sampling week; Hs × Wk = effect of health status by sampling week interaction).

Combined mean concentrations of lactate in the serum were greater in cows affected by lameness *versus* CON cows (4,550 and 2,254 ± 399 μmol/L, respectively; *P* < 0.01; 3). No sampling time effect or health status (Hs) by week (wk) interaction was obtained regarding serum lactate. Interestingly, concentrations of lactate in the serum of cows with lameness were greater than those in the CON cows at all the time points in the experiment (Table 4; Figure 1A), with differences at −8 wks (*P* = 0.04; Table 4) and −4 wks (*P* = 0.04; Table 4) before parturition.

After parturition, both concentrations of NEFA and BHBA in the serum were greater at the week of disease diagnosis in both groups of cows when compared with prepartum means (Figure 1B,C). Sampling week had a pronounced effect on serum concentrations of both NEFA and BHBA (*P* < 0.01; Table 3; Figure 1B,C). At −4 wks before parturition, cows affected by lameness showed a tendency for lower NEFA (*P* = 0.07; Table 4) and greater BHBA (*P* = 0.06; Table 4). But overall serum NEFA and BHBA did not differ between the two groups (*P* > 0.10; Table 3).

**Table 3:** Data of Dry Matter Intake (DMI), milk production, milk composition as well as metabolites, cytokines and APPs in the serum of dairy cows with (n = 6) and without lameness (LAM) during the periparturient period.

	Group ^1^			Effect,^2^ *P*-Value		
Item	LAM	CON	SEM	Hs	Wk	Hs × Wk
DMI ^3^ (kg/d)	17.19	18.64	0.58	0.13	<0.01	<0.01
Milk production ^4^ (kg/d)	36.79	42.16	2.58	0.05	<0.01	0.09
Milk composition ^5^ (g/kg, unless otherwise stated)						
Fat	3.15	3.90	0.14	<0.01	0.04	0.35
Protein	2.86	2.87	0.06	0.94	<0.01	0.45
Fat-to-protein ratio	1.10	1.38	0.09	0.05	0.17	0.63
Lactose	4.55	4.56	0.04	0.84	0.02	0.47
SCC (10^3^ cells/mL)	57.90	30.0	5.37	<0.01	0.10	0.28
Milk urea N (mg/dL)	15.47	15.56	0.96	0.95	0.01	<0.01
TS	12.00	12.19	0.25	0.30	0.03	0.50
Serum parameters ^6^						
Lactate (μmol/L)	4550.67	2254.08	399.21	<0.01	0.20	0.62
NEFA (mmol/L)	261.85	397.16	78.05	0.26	<0.01	0.92
BHBA (μmol/L)	509.38	595.84	66.49	0.39	<0.01	0.35
IL-1 (pg/mL)	287.98	296.66	4.58	0.21	<0.01	0.02
IL-6 (pg/mL)	175.78	26.67	35.51	0.02	0.21	0.34
TNF (ng/mL)	0.40	0.19	0.08	0.09	<0.01	0.03
Haptoglobin (mg/mL)	0.21	0.15	0.03	0.14	0.24	0.03
SAA (ug/mL)	19628	8548.38	2440.73	<0.01	0.02	0.39

^1^ CON = cows without lameness (health control); LAM = cows with lameness. ^2^ Effect of health status (Hs), sampling week (Wk), and health status by sampling week interaction (Hs × Wk). ^3^ DMI was calculated from week −8 to +8 relative to parturition. ^4^ Milk production was calculated from week +1 to +8 relative to parturition. ^5^ Milk compositions were determined on week +2, +3, +5, +7 relative to parturition. ^6^ Serum parameters were calculated from week −8, −4, disease and +4 relative to parturition.

**Table 4:** Data of DMI, milk production, milk composition and serum parameters at the diagnosis week, and concentrations of serum indicators prior to the diagnosis of lameness (LAM).

	8 Week before Parturition			4 Week before Parturition			LAM Diagnosis Week ^1^		
Item	CON	LAM	*P*-value	CON	LAM	*P*-value	CON	LAM	*P*-value
DMI (kg/d)	14.91 ± 0.90	16.49 ± 1.01	0.28	15.90 ± 0.14	15.95 ± 0.67	0.96	20.26 ± 0.89	13.25 ± 1.11	<0.01
Milk production (kg/d)							43.01 ± 1.62	29.78 ± 4.01	0.01
Milk composition (g/kg, unless otherwise stated)									
Fat							5.08 ± 0.45	3.39 ± 0.41	0.02
Protein							3.00 ± 0.10	2.85 ± 0.07	0.25
Fat-to-Protein ratio							1.69 ± 0.12	1.20 ± 0.15	0.03
Lactose							4.54 ± 0.05	4.43 ± 0.13	0.42
SCC (10^3^ cells/mL)							28.33 ± 5.63	66.50 ± 15.31	0.03
Milk urea N (mg/dL)							15.39 ± 0.76	14.08 ± 1.22	0.37
TS							12.21 ± 0.31	13.47 ± 1.21	0.37
Serum parameters									
Lactate (μmol/L)	2455.49 ± 348.63	5427.91 ± 1095.28	0.04	2162.31 ± 184.52	4232.93 ± 748.08	0.04	2,227.61 ± 320.68	4,760.82 ± 519.38	<0.01
NEFA (mmol/L)	140.79 ± 32.77	107.34 ± 20.88	0.41	193.97 ± 47.17	82.89 ± 6.96	0.07	756.51 ± 232.01	594.63 ± 206.20	0.61
BHBA (μmol/L)	351.93 ± 37.71	374.48 ± 31.14	0.65	311.98 ± 18.50	366.32 ± 17.12	0.06	826.91 ± 151.50	586.69 ± 93.88	0.23
IL-1 (pg/mL)	316.79 ± 6.04	293.92 ± 9.23	0.07	320.96 ± 1.59	302.73 ± 7.26	0.05	277.13 ± 5.42	281.30 ± 7.70	0.67
IL-6 (pg/mL)	19.23 ± 5.67	85.37 ± 52.96	0.30	48.24 ± 17.51	250.81 ± 87.08	0.03	23.17 ± 5.18	113.41 ± 21.96	0.02
TNF (ng/mL)	0.34 ± 0.03	0.39 ± 0.12	0.80	0.27 ± 0.05	0.51 ± 0.11	0.10	0.06 ± 0.03	0.48 ± 0.13	0.02
Haptoglobin (mg/mL)	0.19 ± 0.03	0.17 ± 0.01	0.54	0.15 ± 0.01	0.20 ± 0.02	0.05	0.12 ± 0.01	0.36 ± 0.10	0.05
SAA (ug/mL)	8447.67 ± 3373.28	19799.92 ± 4373.22	0.07	3461.25 ± 341.92	9732.1 ± 2625.98	0.03	10401 ± 1722.57	29300.17 ± 8108.13	0.05

^1^ Cows were diagnosed with lameness (n = 6) ranging from week +1 to +3. CON = cows without lameness (healthy control); LAM = cows with lameness.

### 3.2. Cytokines

**Figure 2:**
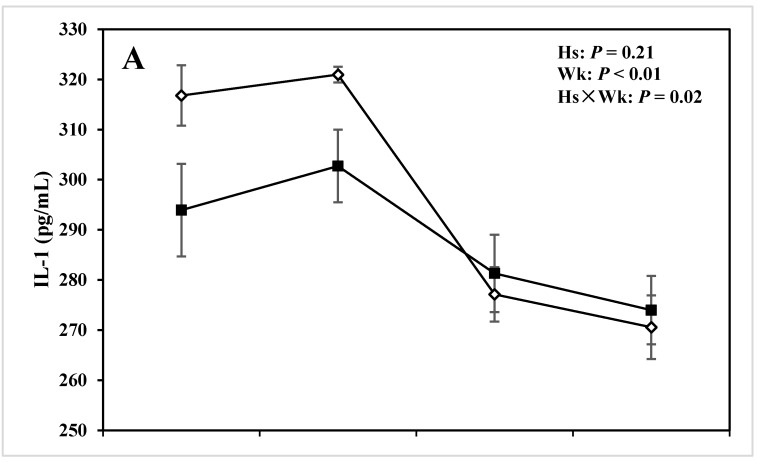

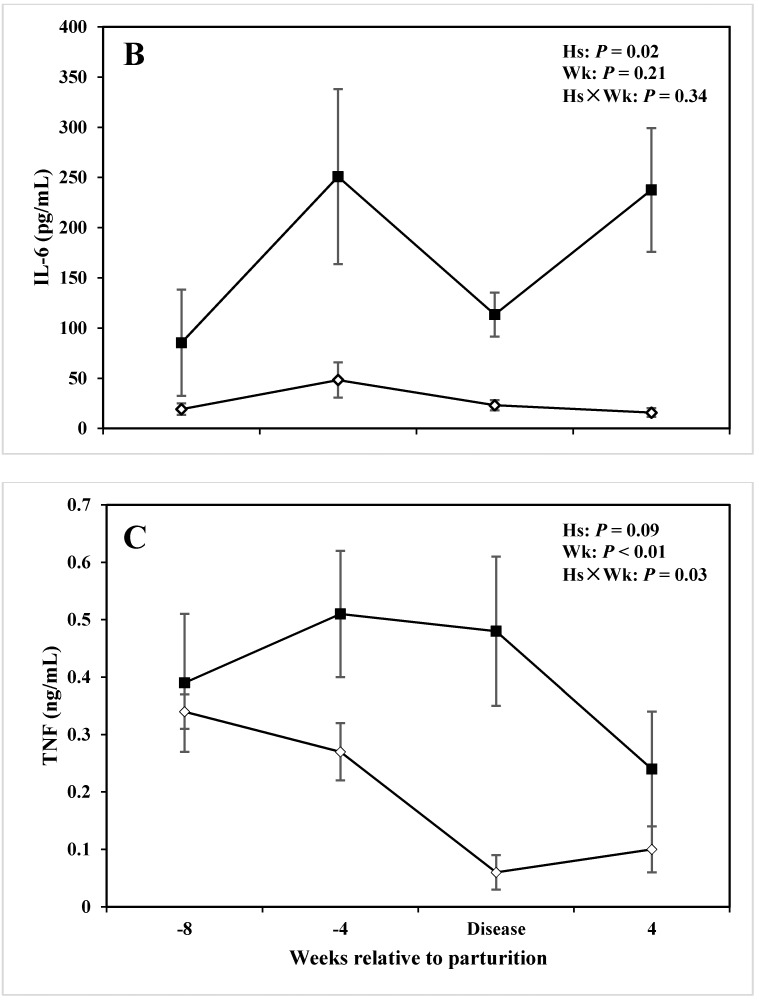
Concentration of (**A**) interleukin (IL)-1, (**B**) interleukin (IL)-6, (**C**) tumor necrosis factor (TNF) in the serum of periparturient dairy cows with (■, n = 6) or without (◇; n = 6) lameness (LSM ± SEM; Hs = effect of health status; Wk = effect of sampling week; Hs × Wk = effect of health status by sampling week interaction).

Cows with lameness had greater concentrations of serum IL-6 throughout the experimental measurements *versus* CON cows (176 *vs*. 27 pg/mL; *P* = 0.02; Table 3). There was a pronounced difference regarding serum IL-6 during disease week (*P* = 0.02; Table 4), and also at −4 wks before calving (250 *vs*. 48 pg/mL; *P* = 0.03; Table 4). There was also a notable decrease in the concentrations of serum IL-6 in both lameness and CON group cows during diagnosis week, where lameness cows showed the lowest concentration of IL-6 in the serum (*P* = 0.02; Figure 2B). No effect of week or the Hs × Wk interaction was evidenced with respect to serum concentration of IL-6.

There was also a tendency of greater concentrations of TNF in the serum in lameness cows compared with the CON ones (0.40 and 0.19, respectively; *P* = 0.09; Table 3). In addition, both Wk (*P* < 0.01; Table 3) and Hs × Wk (*P* = 0.03; Table 3) interaction affected concentrations of TNF in the serum. Particularly, serum TNF decreased at the week of disease diagnosis and concentration continued to decrease to reach the lowest value at +4 wks postpartum in cows with lameness (Figure 2C).

In addition data indicated that Hs did not affect concentration of IL-1 in the serum (*P* = 0.21; Table 3). However, sampling week (*P* < 0.01; Table 4) and Hs by Wk interaction (*P* = 0.02; Table 4) affected concentration of IL-1 in the serum. In addition concentrations of IL-1 were greater before parturition compared with the postpartum values in both groups of cows (*P* = 0.05). There was no pronounced difference between the two groups during the disease diagnosis week (*P* = 0.67; Table 4; Figure 2A). But the results showed that concentrations of serum IL-1 were pronouncedly lower at −4 wks prepartum (*P* = 0.05; Table 4; Figure 2A) and tended to be lower at −8 wks prepartum (*P* = 0.07; Table 4; Figure 2A) in cows with lameness *versus* CON cows.

### 3.3. Acute Phase Proteins

**Figure 3:**
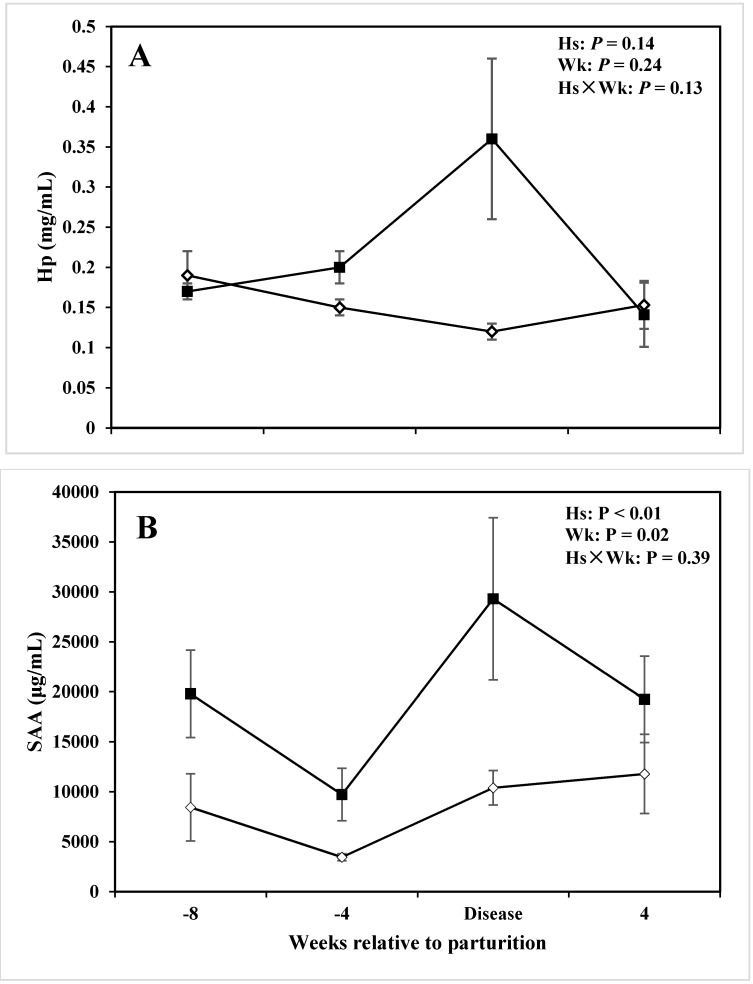
Concentration of (**A**) haptoglobin (Hp), (**B**) serum amyloid A (SAA) in the serum of periparturient dairy cows with (■, n = 6) or without (◇; n = 6) lameness (LSM ± SEM; Hs = effect of health status; Wk = effect of sampling week; Hs × Wk = effect of health status by sampling week interaction).

Statistical processing of the data showed that Hs did not affect the overall mean concentration of Hp in the serum between lameness cows and CON cows (*P* = 0.14; Table 3). Results also demonstrated that there was no effect of Wk on serum Hp (*P* = 0.24; Table 3). However, there was an Hs × Wk interaction effect on the Hp concentration in the serum (*P* = 0.03; Table 3). In particular, concentrations of Hp increased in cows with lameness at the disease diagnosis week, which was almost three times greater than CON cows (0.36 *vs.* 0.12 mg/mL; *P* = 0.05; Table 4; Figure 3A). In addition, this marked difference also was present at −4 wks before calving (*P* = 0.05; Table 4; Figure 3A).

Concentrations of SAA in the serum were greater in cows with lameness *versus* CON ones at all the time points reported (*P* < 0.01; Table 3; Figure 3B). There was also a sampling time effect on SAA in the serum (*P* = 0.02; Figure 3B). The trend of changes of SAA in the serum was almost similar in both groups of cows. More specifically, serum SAA decreased from −8 wks before parturition until −4 wk prepartum. Thereafter, concentrations of SAA increased dramatically when the disease diagnosis was determined after parturition (*P* = 0.05; Table 4; Figure 3B). In Figure 3B, concentrations of SAA in cows with lameness were almost three times greater than CON cows.

### 3.4. DMI, Milk Production and Composition

**Figure 4:**
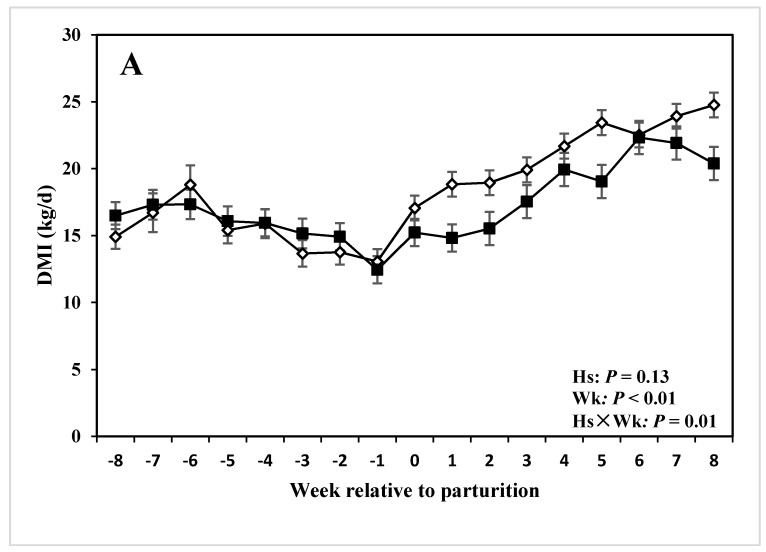

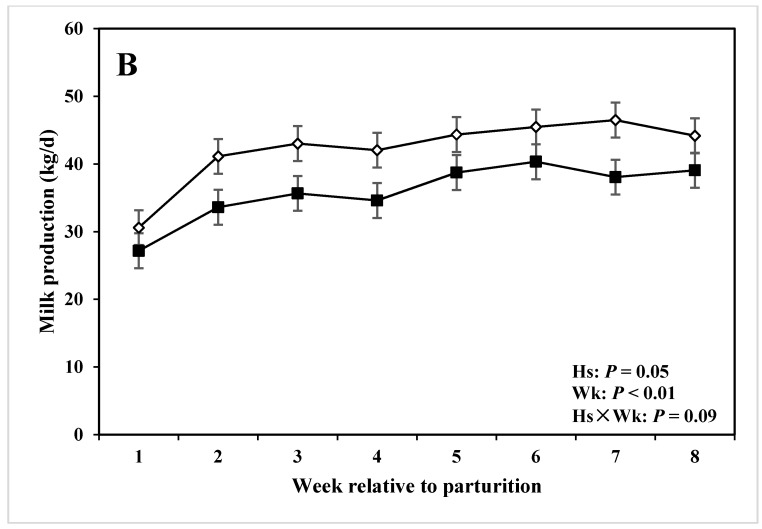
(**A**) DMI, and (**B**) milk production of periparturient dairy cows with (■, n = 6) or without (◇; n = 6) lameness (LSM ± SEM; Hs = effect of health status; Wk = effect of sampling week; Hs × Wk = effect of health status by sampling week interaction).

Cows with lameness tended to have overall less DMI than CON cows (17.19 and 18.64 kg/d, respectively; *P* = 0.13; Table 3; Figure 4). DMI also was affected by the health Hs × Wk interaction (*P* < 0.01; Table 3; Figure 4A). Specifically, DMI was lower for the group of cows with lameness (13.25 ± 1.11 kg/d) compared with those of CON group (20.26 ± 0.89 kg/d; *P* < 0.01; Table 4) in the week when the disease was diagnosed. However, there were no distinctions of DMI between the two groups at −8 and −4 wks prepartum.

Milk yield also was affected by Hs of the cows (*P* = 0.05; Table 3; Figure 4B). The average daily milk yield in the group of cows with lameness (36.79 ± 2.58 kg/d) was lower than CON cows (42.16 ± 2.58 kg/d). Experimental week affected total daily milk production (*P* < 0.01; Table 3; Figure 4B). Moreover, Hs × Wk interaction had a tendency to influence milk yield (*P* = 0.09; Table 3). Daily milk production in cows with lameness was lower compared with CON cows during the disease week (29.78 ± 4.01 and 43.01 ± 1.62 kg/d, respectively; *P* = 0.01; Table 4).

**Figure 5:**
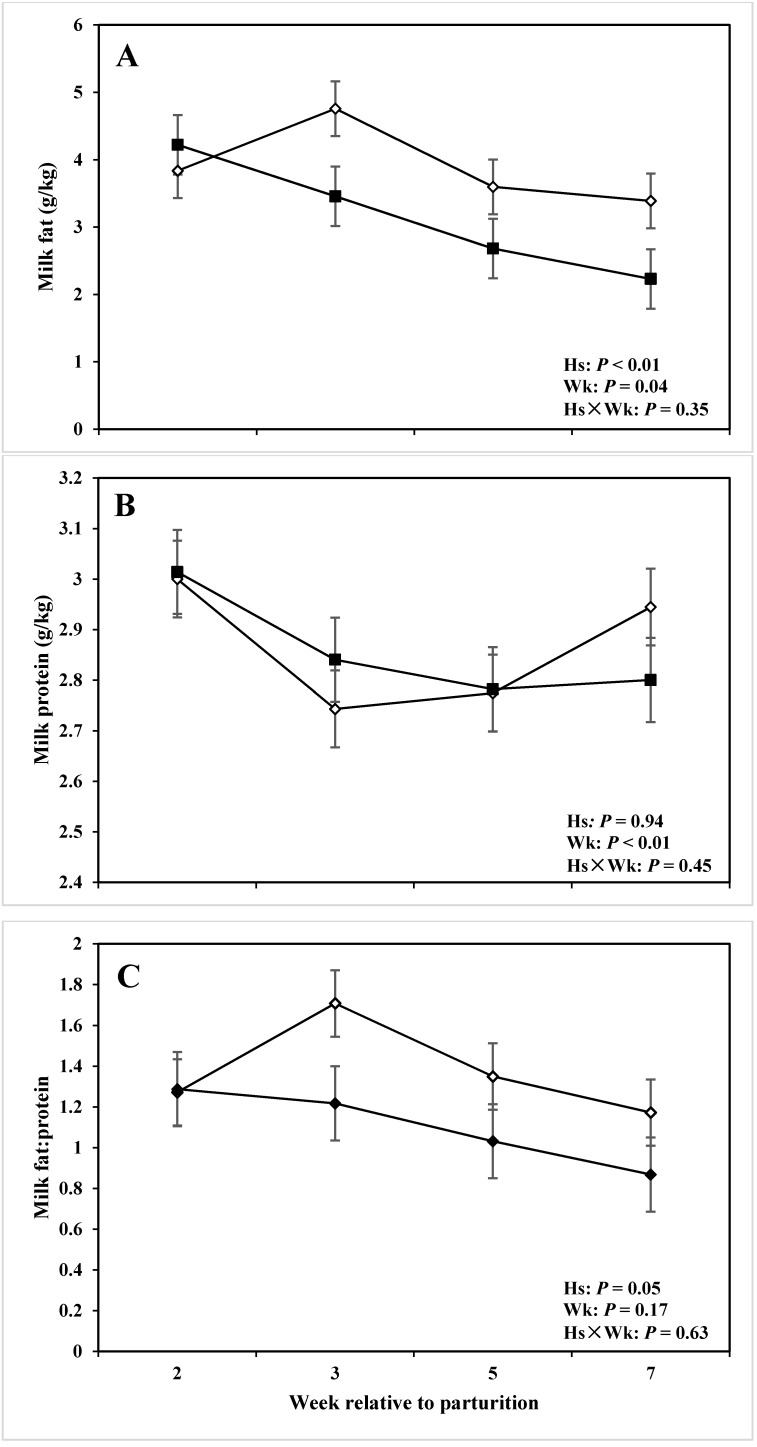
Concentration of (**A**) fat, (**B**) protein, and (**C**) fat-to-protein ratio in the milk of periparturient dairy cows with (■, n = 6) or without (◇; n = 6) lameness (LSM ± SEM; Hs = effect of health status; Wk = effect of sampling week; Hs × Wk = effect of health status by sampling week interaction).

**Figure 6:**
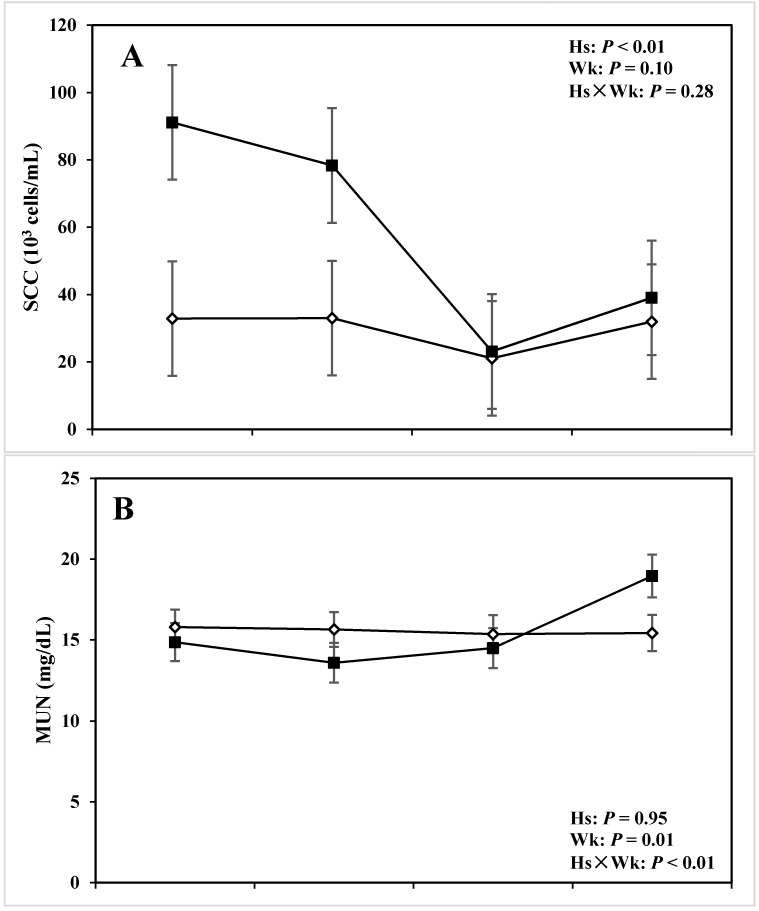

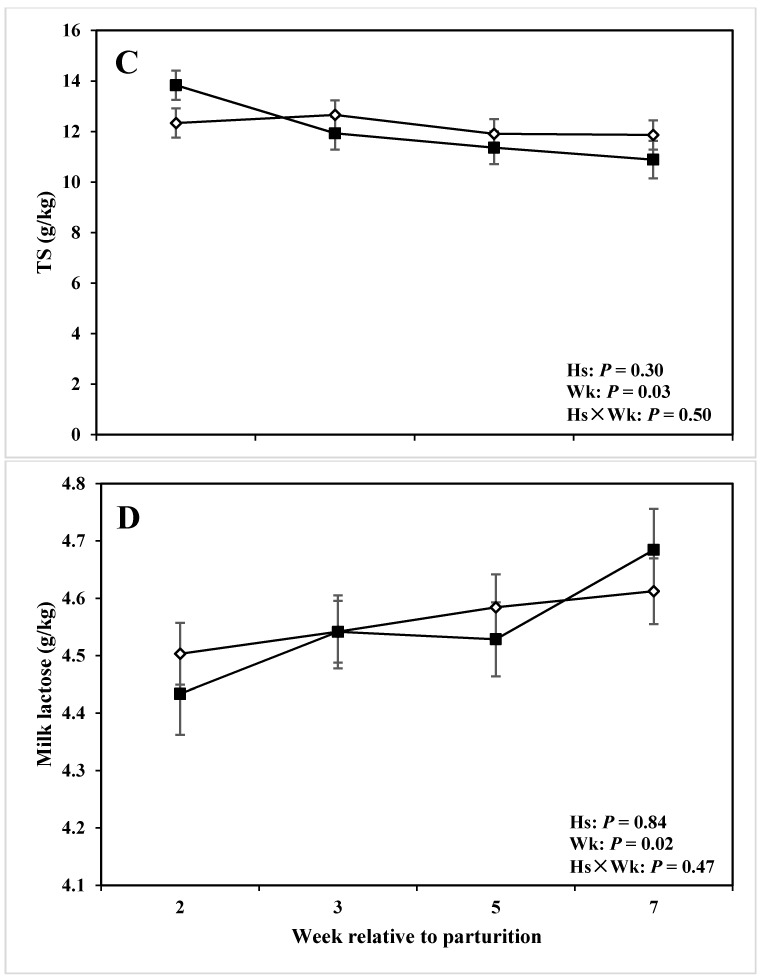
Concentration of (**A**) somatic cell count (SCC), (**B**) milk urea N (MUN), (**C**) total solid (TS), and (**D**) lactose in the milk of periparturient dairy cows with (■, n = 6) or without (◇; n = 6) lameness (LSM ± SEM; Hs = effect of health status; Wk = effect of sampling week; Hs × Wk = effect of health status by sampling week interaction).

The effect of Hs, Wk, and Hs x Wk interaction on milk composition are presented in Table 3 and Table 4 and Figure 5 and Figure 6. Milk fat yield and its ratio with milk protein were lower in cows with lameness than those of the CON group (*P* < 0.01 and *P* = 0.05, respectively) and SCC was greater for cows with lameness compared with CON ones (*P* < 0.01). During the diagnosis week, there was also a difference with respect to milk fat (*P* = 0.02), fat-to-protein ratio (*P* = 0.03), and SCC (*P* = 0.03). There was a tendency for Hs × Wk interaction for MUN (*P* = 0.09). Health status did not affect the amounts of milk protein, lactose, MUN, and TS in this study (*P* > 0.10). However, all tested milk variables were affected (or had tendencies) in relation with the experimental week (*P* < 0.10).

### 3.5. Multivariate Analysis on Serum Variables

**Figure 7:**
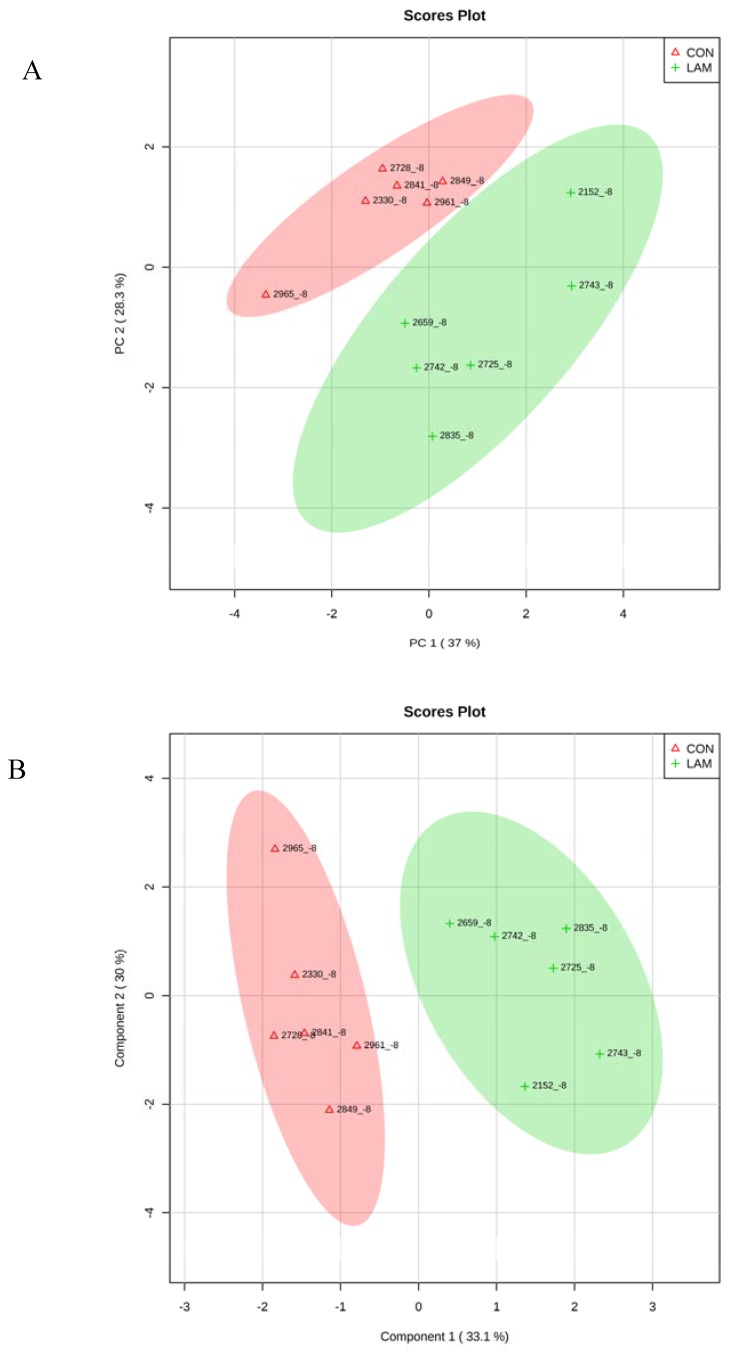
(**A**) Principle component analysis (PCA) and (**B**) Partial least squares-discriminant analysis of six control and six lameness cows at 8 wks before parturition showing two separated clusters for two groups.

**Figure 8:**
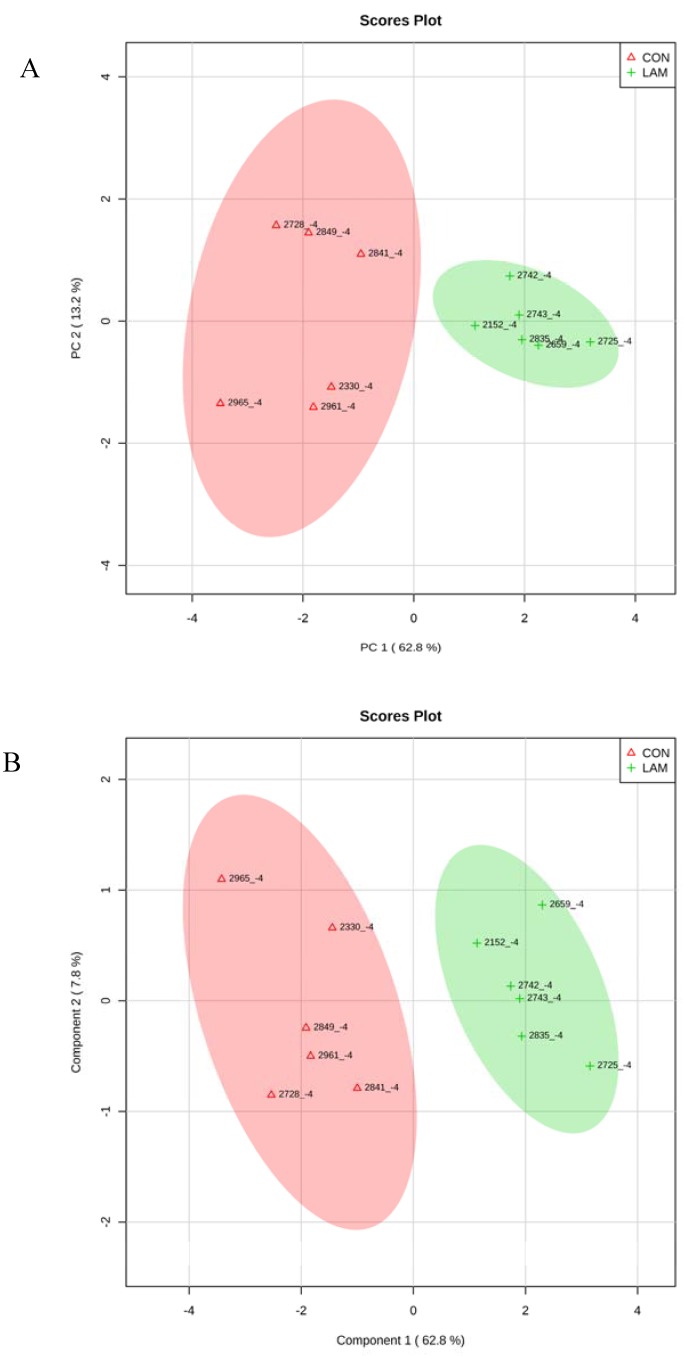
(**A**) PCA and (**B**) PLS-DA of six control and six lameness cows at 4 wks before parturition showing two separated clusters for two groups.

When CON cows were compared with lameness cows at −8 and −4 wk, by PCA and PLS-DA analyses, two clear separated clusters could be seen in both analyses (Figure 7A,B and Figure 8A,B). The results indicated that serum innate immunity reactants and carbohydrate and lipid metabolites profiles between healthy cows and not-yet-lameness cows were already different at −8 and −4 wks before the expected day of parturition.

**Figure 9:**
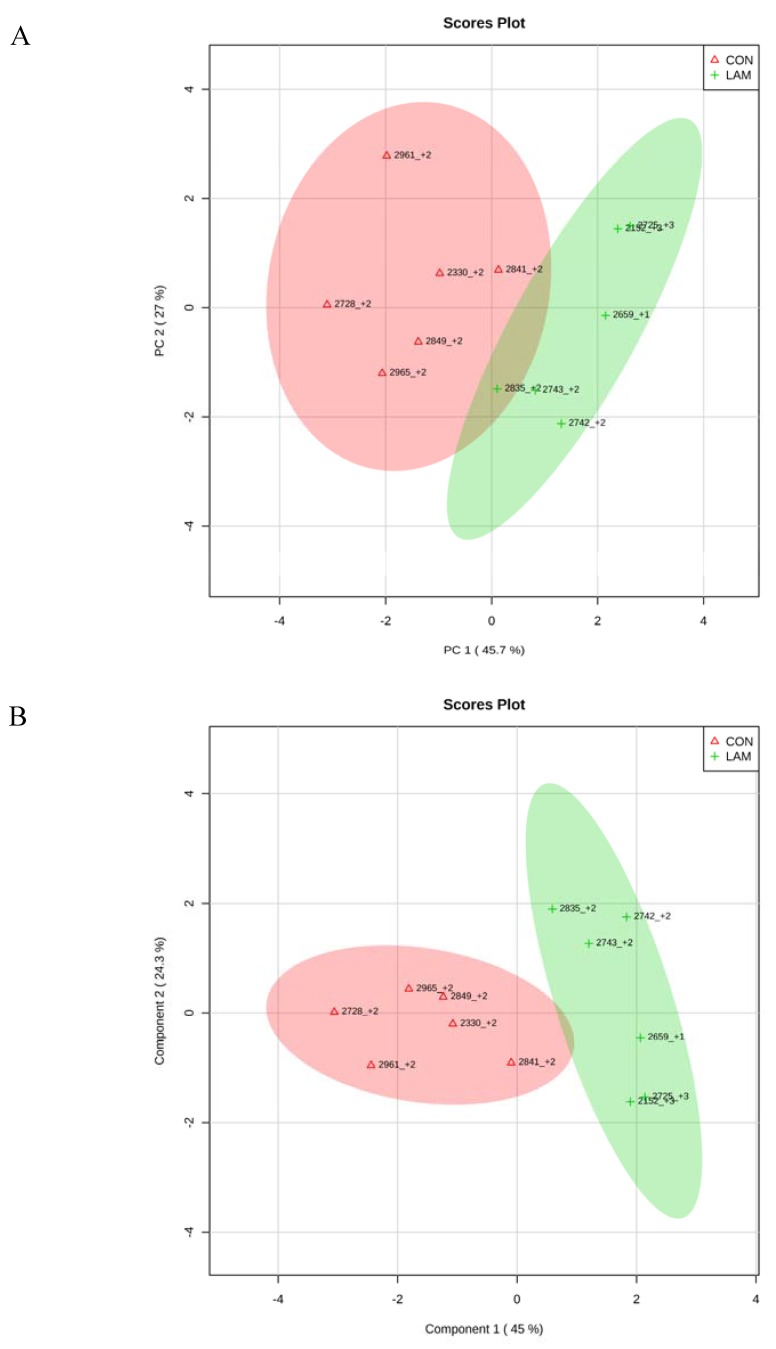
(**A**) PCA and (**B**) PLS-DA of six control and six lameness cows at disease wk showing two separated clusters for two groups.

**Figure 10:**
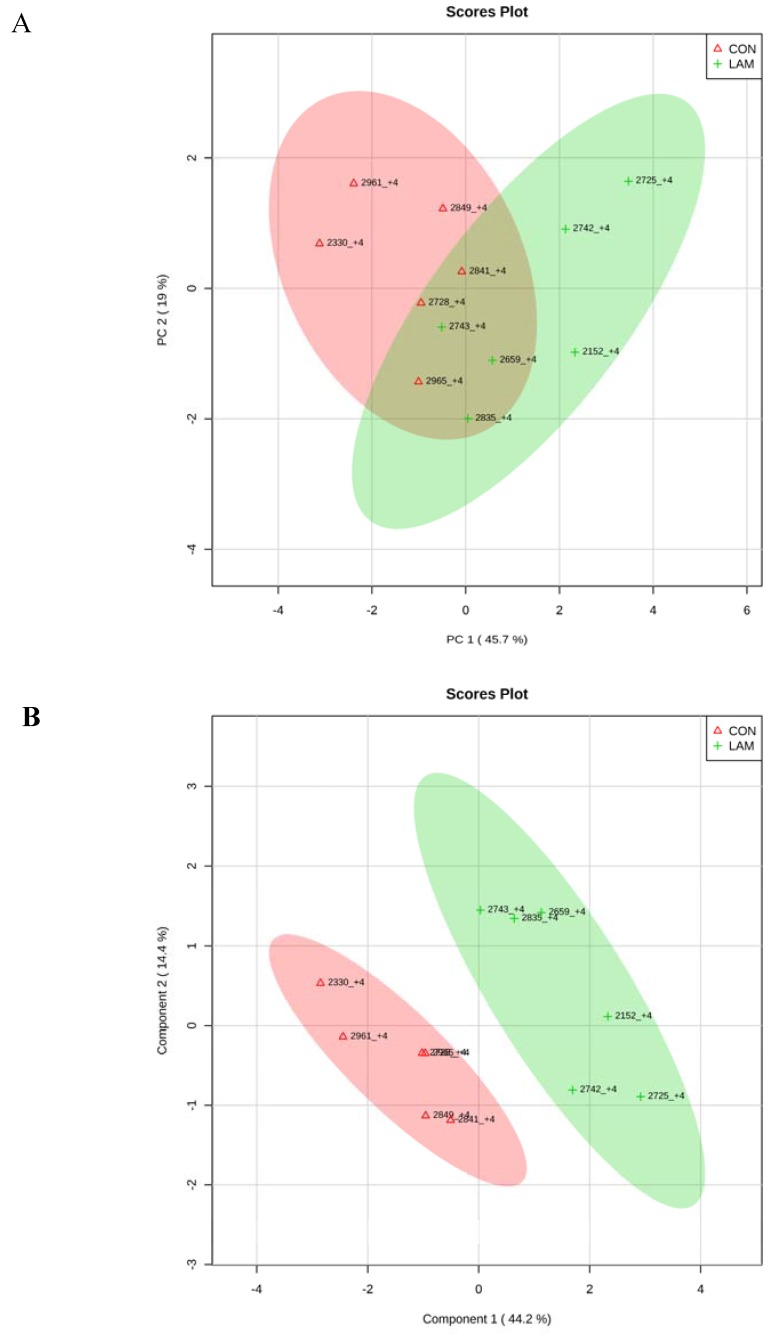
(**A**) PCA and (**B**) PLS-DA of six control and six lameness cows at +4 wk after parturition showing two separated clusters for two groups.

When CON cows were compared with lameness cows at disease diagnosis week, both PCA and PLS-DA analysis revealed a notable and consistent separation between the two groups (Figure 9A,B). Moreover, PCA and PLS-DA also showed a clear separation between healthy cows and cows with lameness at +4 wks after parturition (Figure 10A,B).

### 3.6. Correlation Analysis between Milk SCC and Serum Parameters

Correlations among serum variables and milk SCC are presented in Table 5. Milk SCC showed a positive correlation with lactate, IL-6, and SAA in the serum at all four time points tested with correlation coefficients ranging from +0.70 to +0.92 (Table 5). Furthermore, SCC correlated with serum TNF at −4 wk and at the week of diagnosis of disease (0.80 and 0.84, respectively; Table 5), although the correlations were slightly lower at −8 and +4 wk around calving. In addition, there was a correlation tendency between SCC and serum Hp at −4 wk and disease diagnosis week (0.55 and 0.56, respectively; Table 5). No significant correlations between SCC and serum Hp at −8 and +4 wk was obtained. A negative correlation (−0.76; Table 5) was observed between SCC and IL-1 in the serum at −4 wks prepartum and at −8 wks (−0.53; Table 5) before calving. In contrast, milk SAA did not correlate with serum NEFA or BHBA at all four time points considered (Table 5).

**Table 5:** Pearson’s correlations between milk SCC and serum parameters.

Item		−8 week before parturition ^1^		−4 week before parturition ^2^		LAM diagnosis week ^3^		+4 week after parturition ^4^	
Milk	Serum	r	*P*-value	r	*P*-value	r	*P*-value	r	*P*-value
SCC ^5^	Lactate	0.82	<0.01	0.85	<0.01	0.91	<0.01	0.92	<0.01
	NEFA	−0.04	0.93	−0.33	0.36	−0.11	0.75	−0.36	0.30
	BHBA	−0.11	0.75	0.59	0.10	−0.18	0.62	0.24	0.50
	IL-1	−0.53	0.09	−0.76	0.01	−0.11	0.76	0.11	0.76
	IL-6	0.73	<0.01	0.77	<0.01	0.78	<0.01	0.79	0.01
	TNF	0.47	0.14	0.80	<0.01	0.84	<0.01	0.55	0.12
	Hp	−0.13	0.71	0.55	0.08	0.56	0.09	0.34	0.40
	SAA	0.86	<0.01	0.81	<0.01	0.83	<0.01	0.70	0.02

^1^ Concentrations of serum variables were used from both healthy and lameness cows at −8 wks relative to parturition. ^2^ Concentrations serum variables were used from both healthy and lameness cows at −4 wks relative to parturition. ^3^ Concentrations of serum variables were used from lameness cows at the week of diagnosis of disease and at the same week for healthy cows. ^4^ Concentrations of serum variables were used from both healthy and lameness cows at +4 wks relative to parturition. ^5^ Milk SCC values were used from both lameness and healthy cows at diagnosis week.

## 4. Discussion

We hypothesized that multiple serum biomarkers of lameness could be identified in the transition dairy cows starting at −8 and −4 wks before the expected day of parturition as well as during the week of diagnosis of disease and even during +4 wks after calving. Indeed, results of this study showed alterations in serum concentrations of multiple metabolites as well as proinflammatory cytokines and APPs in cows affected by lameness several weeks before the clinical signs of the disease appeared.

### 4.1. Alterations of Blood Metabolites

One of the most important finding of this study was that concentrations of lactate in the serum were greater in the serum of lameness cows starting at −8 and −4 wks prepartum and became more pronounced at the week when lameness was diagnosed. Lactate has been extensively investigated in veterinary medicine and has been suggested as a useful and practical metabolite to assess severity of illness [27,28]. Previous work has indicated that laminitis-related lameness is highly associated with feeding of diets rich in rapidly fermentable carbohydrates (*i.e.*, high-grain diets) and development of rumen acidosis (*i.e.*, lactic acidosis syndrome) [29,30]; however, this was not the case for cows at −8 and −4 wks before parturition.

Although a remarkable difference of serum lactate between cows with lameness and healthy control started at −8 wks prepartum, DMI between the two groups were not different at −8 and −4 wks before parturition, which suggests that DMI and composition of feed alone could not explain the increased serum lactate concentrations during the prepartal period. Research conducted by our team and others has demonstrated that feeding dairy cattle large amounts of concentrate is associated with increased concentration of rumen endotoxin (a bioactive cell-wall component of all Gram-negative bacteria) and a systemic acute phase response [7,30,31]. Recently, we showed that elevation of serum lactate was strongly correlated to increased rumen endotoxin [32]. However, given the low level of grain feeding at −8 and −4 wks before parturition (*i.e.*, dry-off period), it is not clear what triggers increased lactate in the plasma. It is speculated that presence of dormant pathogenic bacteria (*i.e.*, Gram-negative bacteria) in the mammary gland of the cows affected by lameness might release endotoxin, which stimulates lactate dehydrogenase and increased lactate in the plasma [33]. Lactate dehydrogenase is known to catalyze conversion of pyruvate to lactate. In support of our hypothesis are data that indicate that intravenous infusion LPS in cattle increases plasma lactate [34]. Moreover, the incidence rate of bovine Gram-negative bacterial intramammary infection (IMI) during the dry period is almost three to four-fold greater than during lactation [35].

Concentrations of NEFA and BHBA in the serum are well accepted as indicators of energy balance. During the state of negative energy balance (NEB), both serum levels of NEFA and BHBA increase, which have been correlated with enhanced incidence of disease or impaired reproductive performance [36]. In this study, both serum concentrations of NEFA and BHBA did not show differences between healthy and lameness-affected cows. Enhanced concentrations of NEFA and BHBA in the serum during the disease week and at +4 wks postpartum in both groups illustrated that these cows underwent a state of mild NEB, given the fact that their concentrations were still under the cut-off values suggested for fatty liver or ketosis [37]. DMI data around parturition showed that cows with lameness had lower DMI during the disease week as compared to healthy cows; however, concentrations of NEFA and BHBA around calving did not show a significant difference between the two groups.

### 4.2. Alterations in Innate Immunity

Among the three inflammatory cytokine measured in this study, IL-6 and TNF increased but IL-1 was lower at −8 and −4 wks prepartum in cows with lameness, which suggests presence of a subclinical inflammation. The reason why serum IL-1 was lower prepartum in cows that developed lameness is not clear; however, Fontaine *et al.* [38] reported increased levels of IL-1beta mRNA in perivascular cells of the laminar tissue of horses with induced laminitis suggesting the role of IL-1 in development of a local inflammatory process, and not a systemic one. Interleukin-6, on the other hand, is known to be produced by T helper 2 type (Th2) cells [39]. Previous research has suggested that serum IL-6 can be used as a prognostic biomarker for predicting cows with severe mastitis and prostpartum reproductive diseases like endometritis and retained placenta [39,40]. Our study indicated that concentrations of serum IL-6 were greater in lame cows during prepartum period compared with those after calving, which is in agreement with research conducted by Ishikawa [39]. Furthermore, a greater serum level of IL-6 at −4 wks prepartum was found to affect lameness. Increased TNF in cows with lameness before calving can also be explained by the presence of inflammatory condition and potentially endotoxemic state of those cows. Gabay and Kushner [11] and Emmanuel *et al.* [30] emphasized that translocation of endotoxin into the systemic circulation stimulates the release of pro-inflammatory cytokines such as IL-6, TNF, and IL-1 by liver macrophages, resulting in enhanced secretion of APPs like Hp, SAA, and LBP.

Serum APPs are part of a general nonspecific immune response [41]. Haptoglobin is known for its binding to hemoglobin and antibacterial effects [42], whereas, the functions of SAA and LBP are related to binding, neutralizing, and clearing endotoxin from systemic circulation [43]. Haptoglobin has been used as a biomarker of mastitis [44], metritis [45], and several inflammatory conditions and bacterial infections in dairy cows [46]. Compared with Hp, SAA and LBP have been used as biomarkers of acute diseases [47,48]. Our research showed that all three APPs increased immediately during the week of disease diagnosis (after parturition) compared with the prepartum levels, which confirms previous reports [43]. Enhanced concentrations of serum APPs, like SAA, LBP, C-reactive protein, and Hp are associated with rumen endotoxin and low ruminal pH [30,49]. We did not measure rumen pH or rumen endotoxin in this study.

Furthermore, serum concentrations of all the tested APPs increased already at −4 or −8 wks before calving (prior to occurrence of disease) in cows with lameness than CON cows, which illustrates that Hp and SAA can be considered as potentially early predictors of lameness. Interestingly, there was a decreasing trend for concentrations of SAA at −4 wks prepartum. The reason for this response is not clear. It is speculated that this might be related to involvement of various endotoxin neutralizing factors such as lipoproteins, transferrin, and albumin in removal of endotoxin from systemic circulation as well as increased mucosal barrier functions [11,30].

### 4.3. Milk Production, Composition, and DMI

Data showed that cows with lameness experienced lower DMI and milk production during the experimental period. This can be explained by the effects of lameness on cows’ welfare and wellbeing and the fact that lame cows laid down more than usual and could not consume enough feed to produce more milk [50]. Two other potential mechanisms that might explain the depression in feed intake are subacute rumen acidosis (SARA) and translocation of endotoxin or other potential toxic compounds from ruminal fluid into the systemic circulation [30,31]. In addition, feed intake and milk production were also related to BCS. Cows with greater BCS at calving (>3.5) experience lowered feed intake and milk yield, and an increased risk of metabolic disorders [51].

Another interesting finding of this study was that cows with lameness had milk fat depression and lower fat-to-protein ratio. Lowered percentage of fat in the milk has been associated with decreased rumen pH and SARA [30]. SARA is a trigger for a cascade of events inducing subclinical laminitis as well as other closely related diseases [52]. In recent studies, our group reported strong associations between rumen endotoxin and milk fat depression syndrome in dairy cows [32,53]. Fat-to-protein ratio during early lactation is a helpful indicator of lipomobilization, ruminal acidosis, and periparturinet diseases [52,54]. Several studies have emphasized correlations between fat-to-protein ratio and the incidence of involuntary culling [54,55] as well as occurrence of some postparturient diseases like displaced abomasum, retained placenta, and metritis [54].

Milk fat is the component of milk most affected by a high-grain diet and its effects on rumen fermentation profile [20]. Milk fat content is often used as a predictor of fiber adequacy and the risk of SARA in dairy cattle [56]. Milk fat and its ratio to milk protein were remarkably lower throughout the study, which suggests presence of SARA in the lame cows.

We also found that cows with lameness had greater SCC in the milk, which supports our hypothesis that subclinical mastitis might render cows more susceptible to lameness. In addition, correlation analysis between several serum variables and SCC revealed that innate immune responses in the systemic circulation might be related to inflammation of the mammary gland. Low SCC is used as a reliable indicator of healthy mammary gland and high-quality milk because enhanced milk SCC is mostly related to presence of pathogenic bacteria [57]. In this study, milk SCC showed a positive correlation with serum lactate, IL-6, TNF, and SAA at disease week as well as at −8 to −4 wks before parturition. These results confirm previous reports indicating high incidence of intra-mammary infection during the dry period, which contribute to development of subclinical and (or) clinical mastitis during early lactation [41]. This indicates that the mammary gland of some of the high producing cows is under stress throughout the whole year, including the dry period, rather than during the lactation period alone. This also suggests that screening the health status of the mammary gland throughout the lactation cycle would be a better approach than the routine SCC test during the lactation period. Results of correlation analysis showed that mammary gland might be another source of endotoxins in the blood circulation, besides rumen, in dairy cows. Endotoxin translocated into the systemic circulation might reach the claws directly or induce other agents of disease like biogenic amines that consecutively trigger lameness.

Of note, due to the low number of cows in this study, the findings must be considered preliminary. Further research with a larger number of cows is warranted to elucidate the precise role of prepartum inflammation as well as metabolic and innate immunity responses in the pathogenesis of lameness. Moreover, more research work is warranted to validate the identified blood biomarkers.

## 5. Conclusions

Overall data from this study indicated increased serum concentrations of lactate at -8 and -4 wks before parturition in cows that developed lameness postpartum. Serum lactate has the potential to be used as a predictive and diagnostic biomarker to identify cows that might develop lameness. In addition, concentrations of serum pro-inflammatory cytokines like IL-6 and APPs including Hp and SAA increased at −8 or −4 wks prepartum, preceding development of clinical lameness. Both pro-inflammatory cytokines and APPs can be considered as useful variables to predict and assess the severity of lameness in dairy cows. We also observed that cows with lameness experienced lower DMI, lower milk production, milk fat depression, lower milk fat-to-protein ratio, and greater SCC in the milk during the experimental period. Further studies with larger cohorts of animals are warranted to validate the identified biomarkers.
